# A Cross-Cultural Study of the Cognitive Model of Depression: Cognitive Experiences Converge between Egypt and Canada

**DOI:** 10.1371/journal.pone.0150699

**Published:** 2016-03-24

**Authors:** Shadi Beshai, Keith S Dobson, Ashraf Adel, Niveen Hanna

**Affiliations:** 1 Department of Psychology, University of Regina, Regina, Saskatchewan, Canada; 2 Department of Psychology, University of Calgary, Calgary, Alberta, Canada; 3 Department of Psychiatry, Kasr Al-Aini, Cairo University, Cairo, Egypt; Istituto Superiore di Sanità, ITALY

## Abstract

**Introduction:**

Models of depression that arise in the West need to be examined in other regions of the world. This study examined a set of foundational hypotheses generated by Beck’s cognitive model of depression among depressed individuals in Egypt and Canada.

**Method:**

We recruited 29 depressed and 29 non-depressed Egyptians and compared their results with those of 35 depressed and 38 non-depressed Canadians. Depression status was ascertained using a structured interview, scores on the Beck Depression Inventory, and scores on the Psychiatric Diagnostic Screening Questionnaire. Participants completed questionnaires designed to measure the frequency of negative and positive automatic thoughts (ATQ–N, BHS, and ATQ–P), and dysfunctional attitudes (DAS).

**Results:**

Depressed individuals in both countries had significantly more negative thoughts about self and future, greater frequency of dysfunctional attitudes, and diminished positive self-thoughts in comparison to non-depressed individuals. Egyptians generally showed significantly more dysfunctional attitudes than their Canadian counterparts.

**Discussion:**

The four hypotheses that were tested were supported among the depressed Egyptian sample, which is consistent with the cognitive model. Implications for the cognitive-behavioral model and treatment for this group of sufferers are discussed.

## Introduction

The validity of Western models of psychopathology is often questioned when applied to non-Western individuals. For example, a number of studies have shown that culture may influence the presentation and correlates of depression among individuals of various cultures [1], [2]. The cognitive behavioural model is one of the most popular and widely tested of all models of depression [3], [4], [5], and while it is a Western construction, its hypotheses are stated in universal language [4], [6]. This study examined the extent to which culture moderated the cognitive experience of depression, and whether this cultural moderation rendered the cognitive model less useful in its prediction of the experience of depressed Egyptians. Egypt, which is culturally divergent from Western nations in a number of ways [7] [8], is believed to provide a sound cultural contrast through which the cognitive model of depression and its hypotheses can be examined.

Depression is defined as a condition in which sad mood and/or lack of interest, in addition to other features (e.g., sleep and appetite disturbance, thoughts of worthlessness, suicidal ideation), have persisted for at least two weeks [9]. Depression is highly prevalent in the West and abroad [10]. Okasha [8] found that the lifetime prevalence of depression in urban and rural Egyptian populations to be 11.4 and 19.7%, respectively. Moreover, using the Arabic version of the Diagnostic Interview Schedule, Karam et al. [11] found that the one-year prevalence of major depression in their Lebanese sample was 4.9%. Similarly, Bromet and colleagues [12] reported rates of 10.9% (lifetime) and 4.0% (12-month) of depression in Lebanon.

The cognitive model forwards a number of hypotheses regarding the depressive experience [6], [13]. For instance, Beck and others hypothesized that the differences in cognitions between depressed and non-depressed individuals can be observed in thought access, content, and structure along different levels of the information-processing system. In the deepest and most remote level of the information-processing system, depressed individuals are believed to have depressive schemas or core beliefs (e.g., “I am unlovable”) [6] [13], which can be defined as deep memory representations of the self and world. Given the remoteness of these core beliefs, their presence is inferred from the presence and severity of dysfunctional attitudes and negative automatic thoughts [14]. Dysfunctional attitudes consist of predictions about how the world operates and “if…then” rules for living [15]. As such, the model predicts that, when individuals are depressed, they will exhibit a greater activation of negative schemas, which will be manifest through an increase in dysfunctional attitudes (i.e., activation hypothesis [6]). As predicted by the cognitive model, a number of studies have supported the activation hypothesis and the stability of dysfunctional attitudes, even after depression has remitted [16] [17] [18]. The activation hypothesis has also been supported in various countries around the world [19] [20].

The cognitive model also makes predictions regarding automatic thoughts, which are thoughts that lay just beneath the threshold of awareness. According to the model, negative automatic thoughts (e.g., “no one wants to be around me”) are more frequent among individuals with depression. Further, and in addition to this increase of negative cognitions, Beck hypothesized that depressed individuals would also show lower frequencies of positive thoughts about the self during depression [6] [21]. A recent study [22] found that dysphoric (i.e., showing signs of depression) Egyptian students endorsed more negative thoughts in comparison to students who did not show a marked increase in depressive symptoms. These results were supportive of some earlier research of depressed individuals in the Arab region [23], [24], but are inconsistent with other research [25]. Given the paucity of research examining the cognitive model of depression in this region, the findings obtained by Beshai et al. [22] required replication among a depressed Egyptian sample.

Finally, the model predicts that these cognitive features of depression (e.g., negative thoughts, increased dysfunctional attitudes, etc.) are meaningfully correlated with depression symptoms. In other words, the model predicts that access to and frequency of depressive cognitions increase with increasing depressive symptoms. This is known as the severity hypothesis [6].

These hypotheses are central components of the cognitive model of depression [4]. Cross-cultural examination of these hypotheses is important, as the validity of some central features of the model (e.g., presence of negative thoughts of self) has previously come into question among individuals of Middle Eastern descent [25]. Further, Kazdin [26] emphasized that understanding cultural differences in the presentation and expression of psychopathology can elucidate mediators and mechanisms in psychotherapy research. Furthermore, manualized cognitive therapy for depression assumes the presence of these cognitive correlates of depression, and dedicates substantial therapeutic time to dismantling these cognitive features among patients [15]. As such, examining these central features of the cognitive model among individuals of Egyptian descent is of particular importance, since it may directly inform treatment adaptation efforts in this region.

The current investigation examined whether depressed Egyptian individuals experience fundamental cognitive features of depression much like their depressed Western counterparts. Given the prevalence of depression in the Arab world and the efficacy of the cognitive-behavioural approach in Western countries, it is important to evaluate cognitive-behavioural models and techniques with depressed individuals in the Arab region. Depressed Egyptian and Canadian samples were recruited for the purposes of this study. The results obtained from depressed samples in both nations were directly compared. In addition, two non-depressed samples were recruited in both countries to act as diagnostic controls for the depression condition within each country. In comparison to their non-depressed counterparts in either country, we hypothesized that depressed individuals in Egypt and Canada would exhibit 1) more negative thoughts about the self and future; 2) more dysfunctional attitudes; and 3) fewer positive self-referent thoughts. We also hypothesized that 4) increases in depressive symptoms would correspond to increases in negative cognitions and attitudes, and decreases in positive thoughts.

## Materials and Methods

### Inclusion/Exclusion Criteria

To be included, all participants reported that Egypt (for the Egyptian subsamples) or Canada (for the Canadian subsamples) was the country of birth for self and parents. Canadian participants reported that they were at least third generation European and Egyptian participants reported that they were at least third generation Egyptian. Depressed participants in both countries reported symptoms consistent with a Major Depressive Episode in accordance with DSM-IV-TR criteria [9]. Participants were excluded if they reported symptoms of psychosis, mania, and substance dependence. Non-depressed participants were excluded if they reported a history of depression.

### Participants

Depressed Egyptians were recruited from a psychiatric clinic in Cairo, Egypt. Non-depressed individuals were recruited from the community at large. The clinic from which depressed Egyptians were recruited was located in an affluent Cairo neighborhood. The Egyptian collaborator (AA) was a Christian Egyptian psychiatrist, and the majority of his patients reflected this religious orientation. Depressed and non-depressed Canadians were recruited from the community in Calgary, Alberta, Canada, using poster ads and social media outlets (e.g., Facebook). Participants were compensated with 20 Canadian Dollars, or 100 Egyptian Pounds for participating in the study.

The demographic infromation for study participants can be found summarized in Table 1. Participant age was subjected to a two-way ANOVA, with nationality (Egyptian vs. Canadian) and depression status (Depressed vs. Non-depressed) as between-group variables. This analysis revealed a significant main effect for nationality, *F*(1, 127) = 9.70, *p* = .002, as Egyptian participants were younger (*M* = 30.16, *SD* = 10.05) than their Canadian (*M* = 36.95, *SD* = 14.91) counterparts, regardless of depression status. This analysis also revealed a significant interaction between nationality and depression statuts, *F*(1, 127) = 4.78, *p* = .031, as depressed Canadians (*M* = 41.26, *SD* = 13.45) were significantly older than their depressed Egyptian (*M* = 29.41, *SD* = 8.44) counterparts. Age was found to significantly and positively correlate with hopelessness scores, *r*(129) = .18, *p* = .04. Further, age was also significantly and negatively correlated with positive thoughts about self, *r*(129) = -.25, *p* = .005. Given these results, we used age as a co-variate in the analysis examining differences in main outcome variables.

**Table 1:** Demographic Variables of Depressed and Non-Depressed Individuals, Stratified by country.

Demographics	Depressed (*n* = 64)		Non-Depressed (*n* = 67)	
	Egyptian (*n* = 29)	Canadian (*n* = 35)	Egyptian (*n* = 29)	Canadian (*n* = 38)
Female n (%)	17 (58.2)	23 (65.71)	17 (58.2)	30 (78.95)
Mean Age (*SD*)	29.41 (8.44)	41.26 (13.45)	30.90 (11.54)	32.97 (15.25)
Marital Status *n* (%) Single	21 (72.41)	19 (54.29)	17 (58.20)	22 (57.89)
Married	6 (20.69)	7 (20.0)	12 (41.8)	13 (34.20)
Divorced/Separated	2 (6.90)	9 (25.71)	0	3 (7.89)
Education *n* (%) Secondary	4 (13.79)	9 (25.71)	10 (34.48)	16 (41.11)
Trades/Diploma	4 (13.79)	12 (34.29)	9 (31.03)	8 (21.05)
Bachelor’s Degree	14 (48.28)	9 (25.71)	8 (27.90)	9 (23.68)
Modal Income /Employment Range	Unemployed/No Income (51.70%)	$10,000–30,000 (28.90%)	Unemployed/No Income (55.20%)	$10,000–30,000 (36.8%)
Religious Affiliation *n*(%) Christian	23 (79.31)	20 (57.15)	17 (58.62)	22 (37.89)
Muslim	5 (17.24)	0	12 (41.8)	0
Agnostic/Atheist	0	8 (22.85)	0	10 (26.32)
Other	1 (3.45)	7 (20.0)	0	7 (18.82)

*Note*. Marital Status, Education, and Religious Affilation only represent summaries of the responses. Some subcategories were not included in this table.

Three one-way MANOVAs compared differing levels of economic status (from unemployment to over 100,000 dollars in income), differing levels of education (from no secondary education to a doctorate equivalants), and religious affiliations on the dependent measures of interest (BDI, PDSQ–Dep, ATQ-N, ATQ-P, BHS, DAS). The analyses revealed no significant differences between the various economic statuses (*p* > .05), educational categories, or religious affiliations (*p* > .05) on any of the dependent measures.

### Measures

Structured Interview: The screening process involved a structured interview with 14 (and 4 additional follow-up) “Yes” or “No” questions regarding ethnicity (e.g., “Were you born in Canada/ Egypt”), current depressive symptoms (e.g., “In the last month, has there been a period of time when you were feeling depressed or down most of the day, nearly every day?”), psychosis (e.g., “In the last month, did you hear voices that other people didn’t hear?”), mania (e.g., “Has there been a period of time when you felt so good, “high”, excited, or hyper that other people told you that you were not your normal self?”), and substance abuse (e.g., “Did anyone in your family, friends, a doctor, or anyone else say that you were using alcohol/drugs too much?”). The items were constructed based on DSM-IV-TR [9] criteria for a major depressive episode, psychosis, mania, and substance dependence. Strict cut-offs were enforced and used for inclusion or exclusion from the present study. Research assistants in both Canada and Egypt were trained to use this screen and asked to do so prior to the inclusion of participants in the study. Within the Canadian sample, the 5 depression items in this structured interview evidenced good reliability (*α* = .88). As a partial testament of the interview’s validity, Canadian participants who reported “Yes” to the interview question “In the last month, did you feel sad or depressed for most of the day, nearly every day?” scored significantly higher on the BDI-II than those who answered “No” to this question (*F*(2, 82) = 115.22, *p* < .001).

The Psychiatric Diagnostic Screening Questionnaire: The Psychiatric Diagnostic Screening Questionnaire (PDSQ) [27], [28] is a 125-item self-report inventory in a “Yes” or “No’ response format. The full questionnaire is comprised of 13 subscales assessing 13 common Axis I disorders (e.g., depression, generalized anxiety, panic, social phobia, bulimia, drug and alcohol use, psychosis, health anxiety, agoraphobia, bulimia, posttraumatic stress, and somatization). For the purposes of this experiment, we used the depression, psychosis, drug use, and alcohol use subscales (total of 39 items; psychosis, mania and substance dependence assessments functioned as a secondary screen beyond the initial interview). The depression subscale (PDSQ–Dep) is a 21-item scale designed to screen for the presence and severity of depression. It possesses items that are designed to assess criteria for a mood disorders episode (e.g., “in the last two weeks, did you feel sad or depressed for most of the day, nearly every day”; “did you get less joy or pleasure from almost all of the things you normally enjoyed”; “did you have problems concentrating nearly every day”) in accordance with the DSM-IV-TR. The recommended cut-off for depression is a total score of 9, which provides a sensitivity of .9 and a specificity of .67. The recommended cut-offs for the psychosis, drug, and alcohol use subscales is 1. As the scale does not differentiate between substance “use” and “dependence”, individuals endorsing a score of 1 or higher were asked a number of follow-up questions to ascertain the severity of their substance use behavior.

All 13 subscales of the PDSQ demonstrated excellent test-retest and internal consistency [26]. The scales possessed adequate concurrent validity, as scores were highly correlated with other self-report measures assessing similar constructs. The PDSQ–Dep subscale exhibited excellent Cronbach’s alphas among the Egyptian (*α* = .90) and Canadian (*α* = .95) samples in the current study.

#### Beck Depression Inventory-II

The Beck Depression Inventory II (BDI) [29] is a widely used 21-item self-report inventory designed to measure the presence and severity of depressive symptoms. The scale measures various components of the depressive experience, thus the items capture the affective, cognitive, somatic, and motivational aspects of the disorder. Each item in the BDI consists of 4 statements from which respondents were asked to pick. Such group of statements corresponds to a 4-point Likert scale (from 0–3), thus the range of scores on this instrument is 0–63, with higher scores indicative of depression severity.

The BDI is significantly correlated (*r* = .72) with other known measures of depression, such as the Hamilton Rating Scale for Depression [30] [31], and has been used in many studies with Arabic-speaking populations. For example, Thomas, Campbell, Altareb and Yousif [32] found that the Arabic translation of the BDI exhibited adequate internal consistency, with an alpha coefficient of .82, when used with a student sample in the United Arab Emirates. Another study [33] found that the Arabic-translated BDI evidenced good internal consistency among samples from 18 Arabic-speaking nations, with alpha coefficients ranging from .82 to .93. Finally, Al-Musawi [34] administered the BDI to a student sample in Bahrain and found that it exhibited adequate internal consistency, with an alpha of .83, good test-retest reliability (*r* = .74), and that the BDI successfully differentiated between depressed and non-depressed participants. The BDI possessed excellent Cronbach’s alphas among the Egyptian (*α* = .94) and Canadian (*α* = .97) samples in the current study.

A cut-off score of 14 was used as partial criteria for depression status in the current study. Using this cut-off score among participants from the general population, a study [35] found that the BDI possessed a sensitivity and specificity of 100% and 99%, respectively, and positive and negative predictive values of .72 and 1, respectively. As such, this study utilized this cut-off score, in addition to the responses on the structured interview and a cut-off score of 9 and higher on the PDSQ–Dep, to establish depression status.

#### Automatic Thoughts Questionnaire–Negative

The Automatic Thoughts Questionnaire–Negative (ATQ–N) [36] is a 30-item test designed to assess the frequency of negative automatic thoughts in depression. The items are negatively-valenced and are answered on a 5-point Likert scale (from 1, or “not at all”, to 5, or “all the time”), with scores ranging from 30 to 150 (higher scores corresponding to a greater number of negative thoughts). The instrument instructed respondents to indicate how frequently they thought of the presented statements (e.g., “I am a failure”, “I don’t think I can go on”, etc.) over the last day.

The ATQ–N was found to possess good internal reliability in a previous study (α = .96) [36]. Harrell and Ryon [37] found strong convergent validity for the ATQ–N, as it was shown to significantly correlate with clinician rating of depression, the Minnesota Multiphasic Personality inventory Depression scale, as well as the BDI. Finally, another study [38] found that the instrument discriminated between depressed individuals and those who suffer from other psychopathologies. Beshai, Dobson, and Adel [22] administered the ATQ–N to a sample of Egyptian students and found that it possessed an internal reliability of greater than .90, and that it significantly correlated with the Center for Epidemiologic Studies–Depression scale [39]. In the current study, the ATQ–N exhibited excellent Cronbach’s alphas among the Egyptian (*α* = .98) and Canadian (*α* = .98) samples.

#### Beck Hopelessness Scale

The Beck Hopelessness Scale (BHS) [40] is a self-report measure that consists of 20 “True/False” forced-choice items designed to assess the extent of positive and negative beliefs about the future in the past week. Each item is scored as 1 or 0 (range of 0–20, with higher scores more indicative of hopelessness), and the total score corresponds to the number of pessimistic items that are endorsed.

Beck and Steer [40] found high scale score reliabilities (ranging from .87 to .93) and adequate test-retest reliability (.67) over a one-week period in a psychiatric outpatient sample. The BHS also evidenced adequate concurrent validity, as it has correlated moderately (*r* = .46) with suicidal ideation in a sample of psychiatric outpatients [41]. Finally, the BHS was found to be predictive of suicidal behavior in prospective studies with psychiatric patients [42]. The BHS showed excellent Cronbach’s alphas among the Egyptian (*α* = .94) and Canadian (*α* = .94) samples in the current study.

#### The Automatic Thoughts Questionnaire–Positive

The Automatic Thoughts Questionnaire–Positive (ATQ–P) [43] is a 30-item self-report inventory designed to assess the frequency of positive thoughts such as “I am in a great mood”, or “I have many good qualities” during the past week. The instrument measures frequency on a 5-point scale from 1, or “never”, to 5, or “all the time”. Scores on the ATQ–P can range from 30–150, with higher scores indicating more positive thoughts. Researchers [44] found that the ATQ–P possessed excellent internal reliability, with an alpha coefficient of .95. A study [45] reported that the ATQ–P successfully discriminated between psychopathological vs. non-psychopathological states. These researchers also found that the instrument negatively correlated with the BDI (*r* = -.47) and other measures that assess affective symptoms, thus evidencing adequate discriminant validity. In the current investigation, the ATQ–P possessed excellent Cronbach’s alphas among the Egyptian (*α* = .95) and Canadian (*α* = .98) samples.

#### Dysfunctional Attitudes Scale

The Dysfunctional Attitudes Scale, Form A (DAS) [46] is a 40-item scale originally designed to assess depressogenic schema content. The instrument instructed respondents to indicate their agreement, on a scale of 1–7 (where 1 = “Totally Agree”, and 7 = “Totally Disagree”) to statements such as “I am nothing if a person I love doesn't love me” and “If others dislike you, you cannot be happy”. The negative items are reversed, so scores on the DAS range from 40–280, and higher scores indicate greater negative attitudes. Previous research [47] showed that the DAS has excellent internal reliability, with coefficient alphas ranging from .84–.92. Secondly, the developers [46] found good test-retest reliability for the measure over an 8-week period (*r* = .80). Depressed individuals were found to score significantly higher on the DAS than their non-depressed counterparts in a number of studies [48], [49]. A study [50] found that when the DAS was used among a sample of United Arab Emirates students, the scale possessed adequate internal consistency and concurrent validity, and a factorial structure comparable to those derived from Western samples. In the current investigation, the DAS possessed excellent Cronbach’s alphas among the Egyptian (*α* = .85) and Canadian (*α* = .94) samples.

### Design and Procedure

The University of Calgary’s Conjoint Research Ethics Board approved this study (file 6899). All participants provided written consent prior to their participation in this study. A trained research assistant conducted the structured screening interview with participants either in-person or over the phone. Those who qualified (e.g., no signs of mania, psychosis, or substance dependence, and evidenced depression symptoms in the last month or no depression symptoms or history of depression) were directed to take part in the study. Upon arrival to the clinic/lab, individuals who were deemed eligible on the screen were greeted by a trained researcher and asked to offer their written consent to participate in the study. Those who provided consent were given the first package of questionnaires, which contained a demographic information form, the PDSQ (Depression, Psychosis, Alcohol, and Drug subscales, and Mania), and BDI. Based on scores provided during this section of the study, individuals were prompted to continue the study or were excluded from the study and appropriately debriefed. Scores from participants who were excluded in the first section of the study were excluded from final analyses.

Eligible participants were asked to complete a second questionnaire package, which included ATQ–P, ATQ–N, BHS, and DAS. Following this, all participants were verbally debriefed and provided with a debriefing form which offered more details about the nature of the study as well as a list of mental health resources and materials (e.g., self-help books, in English and Arabic) that could be consulted at the participant’s discretion. Participants were thanked for their participation and given their compensation, as outlined in the advertisement and consent form. Research assistants were trained to assess and document suicidal risk. Participants who showed elevated risk for suicide were strongly encouraged to contact one of the resources provided on the debrief form (e.g., Calgary Distress Centre).

### Translation

All measures used in Egypt were translated in accordance with the World Health Organization’s Guidelines for the Process of Translation and Adaptation of Instruments [51]. These guidelines are harmonious with previously developed guidelines for the translation of self-report medical and psychological measures [52], and stipulated a four step process in the adaptation of instruments: forward translation, expert back-translation, pre-testing, and finalizing. For the purposes of this study, two professional translators (each with a minimum of 5 years of experience) forward translated the original English instruments into Arabic. Subsequently, a third professional translator back-translated the newly formed Arabic instruments to English. The first author (who can speak, read and write both English and Arabic fluently) then compared the back-translated versions with the original instruments and noted discrepancies, and awkwardly worded, or what appeared to be culturally non-equivalent items. After flagging these “difficult” items, the author made a quick translational suggestion for each of these items. Such suggestions guided the forward translators who then retranslated these items to Arabic (with the suggestions in mind), and passed it on for back-translation. This process was repeated a total of three times until all apparent discrepancies from the originals were eliminated from the back-translations.

### Training of Research Personnel

All researchers and research assistants taking part in the study were trained in accordance with a standardized study protocol. The research assistant in Egypt received training via Skype. Further, the research assistant was provided with training videos which provided detailed instructions with respect to the study protocol, and described the nature of the consent and debriefing process, as well as provided descriptions of each of the utilized questionnaires and measures. The research assistant in Egypt was fluent in both English and Arabic, and thus her training and understanding of the instructions (which were in English), as well as her ability to administer the protocol in Arabic, were ensured. The research assistant was a qualified psychologist with over 10 years of experience in mental health.

In addition to soliciting help from research personnel in Cairo, the first author trained research assistants to collect and manage data in Canada. Over the course of the study, six research assistants were recruited and trained. All research assistants were provided the study protocol described above, and were trained over two sessions by the first author with respect to study procedure and risk management. All research assistants involved in the study were asked to practice conducting the screening interview (as well as other portions of the protocol) with two actors prior to live participant recruitment. Assistants’ scores on these mock interviews were recorded and reviewed by the first author. Prior to conducting live interviews, full agreement between the first author’s and the assistants’ scores (on depression related and other eligibility items of the screen) on the mock interviews was required. Further training was provided when deemed appropriate. All research assistants in Canada were either completing or had obtained a Bachelor’s degree in Psychology at the University of Calgary, Canada.

### Participant Selection and Retention

As mentioned above, eligibility for the study followed a two-stage process: initial screening (using the structured interview), and during part one of the study. To be eligible in the first stage, participants were required to show clear diagnostic features of depression (depressed group) or no elevated symptoms of depression (non-depressed group), in addition to meeting the ethinicity and other diagnostic criteria discussed above. In the second stage, to be included in the analyses, participants in the depressed group were required to have total scores of 14 or more on the BDI *and* scores of 9 or more on the PDSQ–Dep. To be included in the analyses, participants in the non-depressed must have total scores below 14 *and* 9 on the BDI and PDSQ–Dep, respectively. In Canada, 233 participants (158 depressed and 75 healthy) were screened prior to taking part in the study. Participants were excluded after this initial screening based on ethinicity (e.g., indicated a birth place other than Canada for self or for parents, or a birth place other than Europe or Canada for grandparents), no current depressive symptoms (e.g., no significant saddness or anhedonia present in the last month), or the presence of other exclusion criteria (i.e., symptoms suggestive of psychosis, mania, or substance dependance). Of those individuals initially screened, a total of 93 participants (39.9%) took part in the study. Of those individuals, 20 were excluded from further analyses based on the secondary screening which took place in the first part of the study. Specifically, six individuals showed signs of psychosis, five showed signs of substance dependance, and nine did not meet criteria for depression on the BDI (i.e., less than 14) and/or PDSQ–Dep (less than 9; yet their scores on one of these measures was elevated beyond the cut-off to be included in the non-depressed sample). A total of 73 participants (35 depressed and 38 healthy) were included in the final analyses of scores on the self-report measures except for on the DAS. One Canadian participant in the depression group failed to complete the DAS, and so their data were excluded from analyses on this measure. Statistical analyses were conducted to examine significant differences on demographic variables between those included in the final analyses and those who were excluded in the second stage. These analyses revealed no significant differences between the groups (*p* > .05) on any demographic variable.

In Egypt, there was a total of 116 participants initially screened, and 58 participants (29 depressed and 29 healthy) who were included in final analyses. Data on excluded participants were not systematically collected, but anecdotal information provided by the research assistant in Egypt indicated that there were very few people who failed to meet the study critieria after the initial screen. Given that the initial screening of the study was done by a liscensed psychiatrist the third author of this study (A.A), it is assumed that the hit rate for inclusion after the screening process was extremely high. Two individuals in the Egyptian (one in each the depressed and non-depressed groups) sample failed to complete the DAS, and so their data for this measure were excluded.

### Statistical Analyses

All data were analyzed using SPSS Version 19.0. Errors, deviant responses, and missing data were examined. Missing data for self-report questionnaires were less than 1%. We used the individual mean imputation to estimate all missing data (each data point was interpolated within each participant, within each diagnostic group and nationality; [53]). All dependent variables were then examined for normality, and reliability estimates for all employed primary outcome measures were calculated for each ethnic group. An *a priori* alpha level of .025 was used for analyses of main hypotheses, as was suggested by Tabachnick and Fidell [54] for parametric tests which violated the homogeneity of variance assumption. All other analyses employed an *a priori* alpha level of .05. First, demographic variables (e.g., age, years of education, income, gender, and marital status) for all groups were analyzed using MANOVA or chi-square analyses.

Secondly, a two-way (Nationality by Depression status) MANCOVA was conducted to compare the groups on the primary outcome variables: PDSQ–Dep, BDI, ATQ–N, ATQ–P, BHS, and DAS, while controlling for age. To test the severity hypothesis, Pearson’s product-moment correlation coefficients were calculated for participants in both cultural groups. For more information, please see data in S1 Data.

## Results and Discussion

### Depression and Negative Thoughts about Self and Future

A summary of means and standard deviations for scores on all self-report instruments is presented in Table 2. The two-way MANCOVA (Depression Status by Nationality) was first used to examine differences on the BDI and PDSQ–Dep. This analysis revealed a significant main effect for depression status on both the BDI, *F*(1, 123) = 748.45, *p* < .001, *η*^2^_p_ = .86, and PDSQ–Dep, *F*(1, 123) = 1201.39, *p* < .001, *η*^2^_p_ = .91, wherein depressed individuals in both cultures scored significantly greater on both depression measures. There was no significant main effect for nationality (*p* > .05). However, there was a significant interaction effect between depression status and nationality on the BDI, *F*(1, 123) = 8.04, *p* = .005, *η*^2^_p_ = .06, and the PDSQ–Dep, *F*(1, 123) = 9.00, *p* = .003, *η*^2^_p_ = .07. Additional t-test analyses revealed that non-depressed Egyptians scored significantly higher than non-depressed Canadians on the BDI, while depressed Canadians scores significantly higher than depressed Egyptians on the PDSQ–Dep.

**Table 2:** Means and Standard Deviations of Depressed vs Non-Depressed Individuals on Depression and other Self-Report Measures Assessing the Primary Outcomes, stratified by Country.

Self-Report Measure	Depressed (*n* = 64)		Non-Depressed (*n* = 67)	
	Egyptian (*n* = 29) *M* (SD)	Canadian (*n* = 35) *M* (SD)	Egyptian (*n* = 29) *M* (SD)	Canadian (*n* = 38) *M* (SD)
PDSQ	11.90 (2.34)	13.51 (3.12)	2.00 (1.77)	1.16 (1.60)
BDI	26.85 (8.94)	31.37 (9.77)	4.46 (3.48)	2.32 (2.29)
ATQ-N	87.27 (30.73)	87.60 (24.46)	45.31 (12.73)	35.00 (8.21)
BHS	10.59 (6.93)	13.00 (4.68)	2.90 (2.72)	2.44 (1.72)
ATQ-P	78.54 (18.61)	61.84 (18.93)	102.65 (20.47)	97.73 (27.54)
DAS	162.15 (35.01)	154.97 (28.17)	134.54 (18.21)	103.23 (23.81)

*Note*. PDSQ = Psychiatric Diagnostic Screening Questionnaire–Depression Subscale; BDI = Beck Depression Inventory–II; ATQ-N = Automatic Thoughts Questionnaire–Negative; BHS = Beck Hopelessness Scale; ATQ- P = Automatic Thoughts Questionnaire–Positive; DAS = Dysfunctional Attitudes Scale.

The two-way (Depression Status by Nationality) MANCOVA examined differences on the ATQ–N and BHS, after controlling for age. This analysis of scores on the ATQ–N revealed a significant main effect for depression status, as depressed individuals scored significantly higher than their non-depressed counterparts, *F*(1, 123) = 169.13, *p* < .001, *η*^2^_p_ = .58. There was no significant main effect for nationality, *F*(1, 123) = 1.56, *p* = .214, and no significant interaction effect, *F*(1, 123) = 2.59, *p* = .11.

Scores on the BHS evidenced a significant main effect for depression status, *F*(1, 123) = 130.40, *p* < .001, *η*^2^_p_ = .52, indicating that depressed individuals scored significantly higher on the BHS than non-depressed individuals. There was no significant main effect for nationality, *F*(1, 123) = 1.18, *p* = .28, and no significant interaction effect between nationality and depression, *F*(1, 123) = 3.03, *p* = .08.

### Dysfunctional Attitudes

The MANCOVA revealed significant main effects for depression status, *F*(1, 123) = 70.51, *p* < .001, *η*^2^_p_ = .36, and for nationality, *F*(1, 123) = 12.64, *p* = .001, *η*^2^_p_ = .09. Depressed individuals in both countries scored significantly higher on the DAS than non-depressed participants, and Egyptian participants scored significantly higher on the measure than Canadians. There was also a significant interaction effect between depression status and nationality, *F*(1, 123) = 7.28, *p* = .01, *η*^2^_p_ = .06, as non-depressed Egyptians scored significantly higher on the DAS than their non-depressed Canadian counterparts.

### Positive Thoughts about Self

The MANCOVA revealed significant main effects for depression status, *F*(1, 123) = 57.67, *p* < .001, *η*^2^_p_ = .32, and for nationality, *F*(1, 123) = 5.95, *p* = .016, *η*^2^_p_ = .05. The analysis demonstrated that depressed individuals in both cultures scored significantly lower than healthy individuals on the ATQ–P, whereas Egyptian individuals generally scored significantly higher on this measure than Canadians. There was no significant interaction between depression status and nationality on the ATQ–P, *F*(1, 123) = 1.41, *p* = .24.

### Relationships of Depression and Cognitions

Table 3 presents Pearson’s product-moment correlation coefficients between scores on self-report depression symptoms questionnaires, and negative and positive cognitions. There were significant correlations among scores on the PDSQ–Dep and BDI, and scores on the ATQ–N, BHS, ATQ–P, and DAS in the expected direction for both the Canadian and Egyptian Samples.

**Table 3:** Pearson’s Product-Moment Correlation Coefficients for Scores on Self-Report Measures, Stratified by Country.

Measure	1	2	3	4	5	6
1. PDSQ	-	.90	.71	.65	-.59	.50
2. BDI	.90	-	.80	.75	-.66	.61
3. ATQ-N	.86	.92	-	.82	-.59	.63
4. BHS	.85	.9	.85	-	-.70	.48
5. ATQ-P	-.58	-.63	-.56	-.68	-	-.42
6. DAS	.69	.75	.80	.76	-.64	-

Note. Coefficients for Canadian participants are presented below the diagonal, whereas coefficients for Egyptian Participants are presented above the diagonal. All correlations are significant at the p < .01 level.

Note. PDSQ = Psychiatric Diagnostic Screening Questionnaire–Depression Subscale; BDI = Beck Depression Inventory–II; ATQ-N = Automatic Thoughts Questionnaire–Negative; BHS = Beck Hopelessness Scale; ATQ-P = Automatic Thoughts Questionnaire–Positive; DAS = Dysfunctional Attitudes Scale.

When correlations were significant among participants of both nationalities, Fisher’s r-to-z transformations were conducted in order to compare statistical differences in the degree of association between depressive symptoms and the variable of interest. The first Fisher’s r-to-z transformation compared the nationalities on the correlation of PDSQ–Dep and ATQ–N scores. This analysis revealed a significantly stronger correlation between scores on these measures among Canadians when compared to Egyptians (*p* = .02). The second transformation compared the correlation between PDSQ–Dep and BHS scores. This transformation also revealed a significantly stronger relationship among these variables in the Canadian sample, *p* < .001, when compared to this correlation among Egyptians. There was no significant difference between the samples in the degree of association between scores on the PDSQ–Dep and ATQ–P, *p* > .05. There was no significant difference between the samples in the degree of association between scores on the PDSQ–Dep and DAS.

A Fisher’s r-to-z transformation revealed a significant difference between Egyptians and Canadians in the degree of association of BDI and ATQ–N scores, wherein Canadians’ scores on these measures were significantly more correlated than among Egyptians, *p* < .05. Further, there was a significantly higher correlation among Canadians in comparison to Egyptians between scores on the BDI and BHS, *p* < .01. Finally, there were no differences among the samples in the degree of association between BDI and ATQ–P, and BDI and DAS scores, *p* > .05.

The current investigation examined four core hypotheses of the cognitive model of depression. This cross-sectional study used a two (nationality) by two (depression status) group design in order to examine differences in the cognitive profiles of depressed Egyptians in comparison to their non-depressed counterparts, and depressed and non-depressed Egyptians in comparison to depressed and non-depressed cultural control groups in Canada. As predicted, depressed groups in both countries scored significantly higher than non-depressed individuals on self-report measures of depressive symptoms.

Depressed individuals in Egypt scored significantly higher than depressed individuals in Canada on the PDSQ–Depression subscale, but the difference of 1.61 points is likely not clinically significant. Furthermore, non-depressed individuals in Egypt scored significantly higher than their Canadian counterparts on the BDI. Despite this difference, non-depressed Egyptian individuals’ mean score was still well below suggested cut-offs for minor depression [33]. Further, the relatively elevated depression scores among non-depressed Egyptians may have been due to the political and social unrest that has affected the country during the study period (from 2010 to 2014). Political and social unrest are often associated with elevations in depressive symptoms [1] [55].

As predicted, depressed individuals in both Egypt and Canada exhibited significantly more negative thoughts about the self and future, as were measured by the ATQ–N and BHS (even after controlling age, which was found to covary with BHS scores), respectively. These results substantiate the negativity hypothesis generated by the cognitive model among this sample of depressed Egyptians. The experience of negative thoughts typifies depression in Western nations [4] [6]. Some researchers have found that negative thoughts about the self (or self-reproach) and future are not frequently experienced features of depression in the Islamic and Arabic regions (see Beshai, Dobson, & Adel [22] for review). This pattern of results has led some to conclude that negative thoughts that often accompany affective disorders in the West are rare in non-Judeo-Christian patients [25]. However, and consistent with results obtained with a sample of dysphoric Egyptian students [22], Egyptians suffering from depression were as likely as their Canadian counterparts to experience self-reproach, guilt, and hopelessness.

Depressed individuals in both Egypt and Canada showed a significantly lower frequency of positive self-referent thoughts in comparison to their non-depressed counterparts. There was also a main effect for nationality, wherein Egyptians showed significantly higher scores on the measure of positive thoughts than did Canadians. This finding is inconsistent with other research on positivity [56]. However, most cross-cultural studies on positivity have been conducted with East Asian participants.

As predicted, depression severity was significantly associated with negative and positive thoughts, and dysfunctional attitudes among both Canadians and Egyptians. This pattern of results gives credence to the severity hypothesis among sufferers of Egyptian decent. Further, this pattern gives support to the convergent validity of the measures utilized among Egyptians in the present study. Furthermore, the performed Fisher’s r to z transformations revealed that depressive symptoms may be differentially associated with negative cognitions. This pattern of results is consistent with findings from studies with Asian participants, which showed that the relationship between negative affect and negative health consequences is moderated by culture [57]. In the current study, although depression was found to strongly correlate with negative cognitions in both cultures, as is predicted by the cognitive model, this degree of association was found to be stronger among the Canadian sample.

The present study found that depressed individuals in both cultures endorsed a significantly higher score on a measure designed to assess dysfunctional attitudes in depression (i.e., the DAS). In line with the cognitive model, heightened depression symptoms among Egyptians seemed to be associated with a preponderance of negative self-referent attitudes [6].

Unexpectedly, significant cross-national differences emerged on scores of dysfunctional attitudes, wherein Egyptian participants endorsed significantly more dysfunctional attitudes than their Canadian counterparts. Further, there was a significant interaction effect, as non-depressed Egyptians endorsed significantly more dysfunctional attitudes than their non-depressed Canadian counterparts. This may be accounted by the finding that Islamic societies emphasise modesty and humility, and so may seem self-effacing or devaluing of their own accomplishments when tested by Western measures [58] [59].

### Strengths and Limitations

This was one of the first studies to directly examine hypotheses derived from the cognitive model of depression in an Egyptian sample, and so addressed a huge gap in the literature. A major strength of the current design is the recruitment of a Western sample to act as a parallel cultural control. Most studies in the field of cross-cultural psychopathology collect data from one target country and compare such data to extant literature arising from the West. Further, the current study used common measures in the depression literature, and thus continuity between the results of this study and previous research is possible. Moreover, the measures used have established psychometric properties, and so the obtained results are not likely due to errors in instrumentation. Furthermore, a rigorous and systematic process was followed in order to translate the English measures into Arabic. As a testament of the success of this process, the English and Arabic versions of the scale demonstrated almost identical Cronbach’s alphas and inter-measure correlations.

Despite these strengths, the study had several limitations that potentially affected its validity. First, there were differences in the recruitment strategies utilized in both countries; whereas depressed Canadians were recruited from the community, depressed Egyptians were recruited from a psychiatric clinic. These differences in recruitment strategies across countries may have correlated with other important variables that have not been systematically measured, and thus these differences may have unduly influenced the study results. Similarly, there were demographic differences recorded between depressed and non-depressed, as well as Egyptian and Canadian participants. For example, the Egyptian sample had a higher socioeconomic status and had a higher proportion of Christians than is expected of the Egyptian population [8]. As such, the sample collected in Egypt may not have been fully representative of that population. Further, extrapolations about depressed Egyptians were made from data arising from of other Middle Eastern cultures (e.g., Lebanon). This may be problematic, as there are a number of differences between Egyptian culture and other Arabic and Middle Eastern cultures, and as such, the psychosocial constructs discussed in this paper may not be identical across these nations. However, given the paucity of this type of research and the need for empirical grounding, it was at times necessary to cautiously generalize from findings of previous studies conducted in Arab countries.

Another limitation of the current design was the reliance on cut-offs on self-report measures to establish a status of depression. As Ingram and Siegle [60] indicate, depressed individuals appear to have both quantitatively and qualitatively different experiences than healthy individuals. As such, diagnostic decisions made on the basis of self-reports may suffer from weaker sensitivity and specificity in comparison to the gold-standard, structured clinical interviews. However, it is likely that the majority of cases and non-cases identified in this study were accurately represented, given the fact that the current design used cut-offs on two self-report measures as opposed to just one, and relied on a structured clinical interview to assess presence of current depressive symptoms, which was later corroborated by data from the self-report measures. Further, and although the Diagnostic Interview Schedule [61] has been translated into Arabic, the Structured Clinical Interview for the DSM-IV [62], which is considered the gold-standard for depression diagnosis, does not have an Arabic translation. Another limitation is that the cut-offs that were used for some self-report measures used in this study (e.g., PDSQ) have not been established among Egyptians. Finally, we relied on the Western (DSM-IV-TR) definition of depression in this investigation. This is problematic, as there may be differences in how depression is defined in Egypt and other regions around the world [1]. Moreover, this was a cross-sectional study, and therefore it is difficult to infer causality or directionality in the observed results. Further, the exclusive reliance on self-report measures may have inflated the reported effect-sizes.

### Future Directions

Mental health research efforts that focus on depression have been lacking in the Middle East, despite its prevalence in that region; most depression research that has been conducted in the Middle East is still largely descriptive in nature, and focuses only on epidemiology and symptomatology. As such, more research is needed to increase our understanding of this disorder among individuals of Middle Eastern descent. Future efforts should further establish the nomological network of depression [63]. This work will require attention to the adaptation of extant measures, as well as the creation of new measures that may be more appropriate for use with this population of sufferers [64]. Further, and once the psychometric groundwork is laid in this region, other hypotheses related to the cognitive model need to be examined. For instance, causal and cognitive vulnerability hypotheses (such as the stability of negative schemata; sociotropic and autonomic personality diathesis; etc.) have seldom been tested outside of the West [6] [65]. Finally, validation of other hypotheses forwarded by the cognitive model of depression among Egyptians is in order. For example, a careful examination of the selective attention and memory hypotheses is warranted. Further, casual links between attention and depression need to be replicated among individuals of Egyptian descent [66] [67].

Subsequent to the establishment of the cognitive theory, future studies should tailor the extant cognitive treatments and pilot test their efficacy and acceptability among patients in the Middle East. For example, the tailored cognitive treatments for Middle Eastern sufferers of depression may infuse Islamic elements in the treatment manual, even while they preserve basic principles of cognitive mediation [59]. Pilot testing and feasibility trials should be followed by larger, more definitive randomized controlled trials to establish the efficacy of these tailored treatments among this target subpopulation of sufferers, especially when benchmarked against standardized treatments [68]. Future research should isolate moderators (e.g., culture or other demographic variables) and mediators (e.g., cognitive change) [69] of outcome in the treatment of depression. These studies can then help to build treatment algorithms to support the case conceptualization process in psychotherapy for optimized efficacy. Future research should also isolate the active ingredients which make these types of treatments more effective for clients with a more severe presentation. Finally, and after the validation of cognitive therapy is substantiated among individuals of other cultures, dissemination efforts are warranted. This is likely to be challenging, as dissemination and uptake of evidence-based psychotherapies remains stunted in Western nations [70], let alone among developing countries.

### Conclusions

Overall, the findings of the present study are supportive of the cognitive model with sufferers of that region. For instance, both depressed individuals in Egypt and Canada displayed more negative automatic thoughts (toward self and future), more dysfunctional attitudes, and fewer positive self-referent thoughts in comparison to their non-depressed counterparts. Further, depressive symptoms were significantly and meaningfully associated with these cognitive concomitants of depression among the Egyptian sample.

Despite this overall pattern of results, a few inconsistencies with the model were obtained. In addition to further studies of the nature, vulnerability, and course of depression in the Middle East, future endeavours should also focus on using basic research in order to isolate consistent predictors of outcome in depression treatments. This research, which has only just begun in Western societies, can be used to tailor treatments to make them more efficacious and appropriate for subpopulations of sufferers at home and abroad. Efforts such as ones described here are encouraged in order to provide better access to good treatments for depression for the millions of sufferers around the world.

## Supporting Information

S1 Data Data from Beshai et al. Cognitive Model of Depression in Egypt–Total Scores. (ZIP)
